# Polymeric nanoparticle-docetaxel for the treatment of advanced solid tumors: phase I clinical trial and preclinical data from an orthotopic pancreatic cancer model

**DOI:** 10.18632/oncotarget.12668

**Published:** 2016-10-14

**Authors:** Si Yeol Song, Kyu-pyo Kim, Seong-Yun Jeong, Jin Park, Jaesook Park, Joohee Jung, Hye Kyung Chung, Sa-Won Lee, Min Hyo Seo, Jung-shin Lee, Kyung Hae Jung, Eun Kyung Choi

**Affiliations:** ^1^ Department of Radiation Oncology, Asan Medical Center, University of Ulsan College of Medicine, Seoul, Korea; ^2^ Department of Oncology, Asan Medical Center, University of Ulsan College of Medicine, Seoul, Korea; ^3^ Institute for Innovative Cancer Research, Asan Medical Center, Seoul, Korea; ^4^ Asan Institute for Life Sciences, Asan Medical Center, University of Ulsan College of Medicine, Seoul, Korea; ^5^ College of Pharmacy, Duksung Women's University, Seoul, Korea; ^6^ Korea Institute of Radiological and Medical Sciences, National Project to Establish Platform to Develop The New Concept Therapy, Seoul, Korea; ^7^ Department of Parenteral Delivery Program, Samyang Pharmaceuticals R&D, Daejeon, Korea

**Keywords:** PNP-DTX, phase I, MTD, efficacy, pancreas

## Abstract

We assessed the efficacy of the polymeric nanoparticle containing docetaxel (PNP-DTX) in preclinical mouse models and determined the maximum tolerated dose (MTD) through clinical study. Subcutaneous and orthotopic mouse models were dedicated. Tumor growth delay in orthotopic model and quantification of *in vivo* imaging in orthotopic model were evaluated. Phase I clinical study was a single-center, prospective, open-label trial in advanced solid tumors. PNP-DTX was injected intravenously and the starting dose was 20 mg/m^2^ escalated to 35 mg/m^2^, 45 mg/m^2^, 60 mg/m^2^ and 75 mg/m^2^. Pharmacokinetics, tumor response, toxicities were evaluated. Preclinical result revealed the more potent cytotoxic effect of PNP-DTX than docetaxel (DTX). However, there was no difference between PNP-DTX and DTX in subcutaneous model. Tubulin polymerization assay showed that PNP-DTX preserved original mode of action of DTX. For phase I clinical trial, 18 patients were analyzed. The dose of 75 mg/m^2^ was tentatively determined as the MTD and the most common toxicity was grade 4 neutropenia not lasting over 7days. The C_max_ of 60 mg/m^2^ PNP-DTX and AUC_last_ of 45 mg/m^2^ PNP-DTX were measured to be comparable to those of 75 mg/m^2^ DTX. Partial remission (PR) was achieved in 4 (22%) patients. The potency of PNP-DTX was revealed especially in orthotopic mouse model. The MTD of PNP-DTX could not be confirmed, but 75 mg/m^2^ was tentatively determined. The PNP-DTX of 45 mg/m^2^ had the same pharmacokinetic profile with that of 75 mg/m^2^ DTX.

## INTRODUCTION

Docetaxel (DTX) is widely used as a chemotherapeutic agent in single or combined regimens for breast, gastric, ovarian, non-small cell lung, head and neck, and prostate cancers. Its anti-neoplastic mechanism is mitotic spindle inhibition via binding to microtubules, resulting in stabilization of the microtubules and spindle [1, 2]. Despite the fact that DTX is an effective anticancer agent for the treatment of a broad spectrum of cancers in various stages with distinct chemical entities, it also has some cytotoxic adverse effects [3]. In a clinical setting, the most severe non-hematological adverse effects by any taxane cytotoxic agent are peripheral neurotoxicity and hypersensitivity [4].

Polymeric nanoparticle (PNP) is a challenging but well-known controlled drug delivery method that exploits the Enhanced Permeability and Retention (EPR) effect of water-insoluble drugs into tumors [5]. Oncologists expect to use PNP-targeted drug delivery to improve the anticancer effects of drugs on neoplastic cells and decrease their adverse effects on normal tissue. However, the biodegradability of PNP is important for the safety of this drug, because a PNP is also an exogenous material that can induce toxicity by its chemical properties.

Several years ago, we developed a new formulation of biodegradable and stable PNP suitable for loading water-insoluble drugs (e.g., taxane cytotoxic agents including DTX) and reported development of PNP containing DTX (PNP-DTX) antineoplastic agent [6]. After demonstrating the stability and constant cytotoxic effects of PNP-DTX, we performed a preclinical study using an orthotopic mouse model of pancreatic cancer to expand clinical indication of the PNP-DTX. We also performed a clinical study to translate its preclinical result into clinics.

We demonstrated the efficacy of PNP-DTX in pancreatic cancer through a preclinical study in an orthotopic mouse model. In addition, in our present phase I clinical trial, we determined the maximum tolerated dose (MTD) and dose-limiting toxicity (DLT) of PNP-DTX, and evaluated its safety and efficacy.

## RESULTS

### Efficacy of PNP-DTX in preclinical mouse models

**Figure 1:**
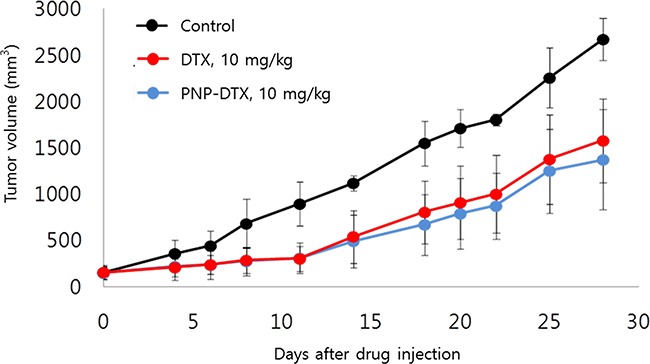
Tumor growth delay in subcutaneous mouse model

**Figure 2:**
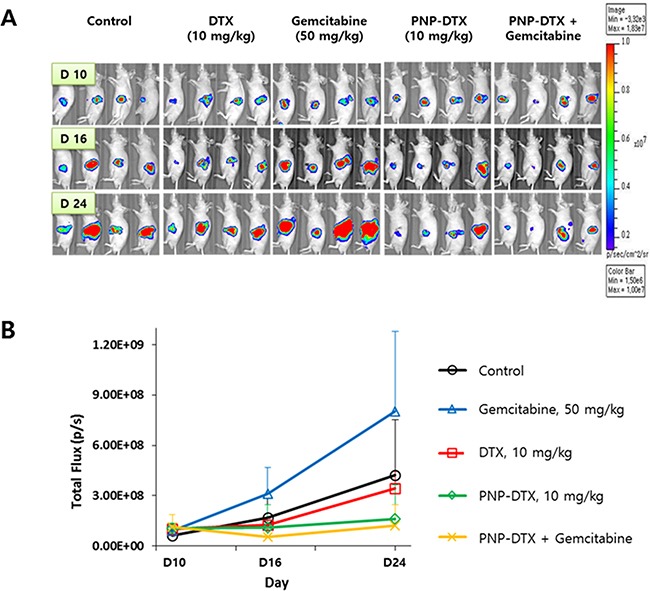
Preclinical efficacy of PNP-DTX in orthotopic mouse model **A.** *In vivo* luminescence imaging after injection. **B.** Quantification of luminescence by live image software.

Tumor growth delay was measured *in vivo* in a subcutaneous mouse model with the BXPC3 cell line. Both PNP-DTX and PNP delayed tumor growth and a minimally increased potency was observed in the PNP-DTX group (Figure 1). In *in vivo* imaging using orthotopic mouse model, PNP-DTX was found to be more potent than DTX or gemcitabine (Figure 2A). Gemcitabine, which can be clinically applicable to pancreatic cancer, was also tested to compare the efficacy in orthotopic model. Quantitative analysis was also done to measure the luminescence (Figure 2B). PNP-DTX had cytotoxic effect both in subcutaneous and orthotopic BXPC3 pancreatic cancer model, while DTX had little cytotoxic effect in orthotopic model. Gemcitabine did not show effect as a single agent in orthotopic model either.

**Figure 3:**
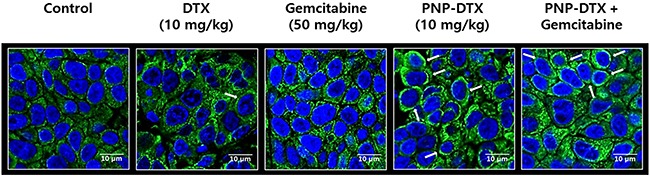
Tubulin polymerization assay visualizing condensation of microtubule (white arrows)

Tubulin polymerization assay was done to know the mode of action (MOA) of PNP-DTX (Figure 3). Both DTX and PNP-DTX showed stabilized microtubule assembly and then thickened cytoplasmic skeleton, while gemcitabine had no morphological change compared with control. This means that PNP-DTX continues to have its original property of DTX.

### Patient Characteristics

**Figure 4:**
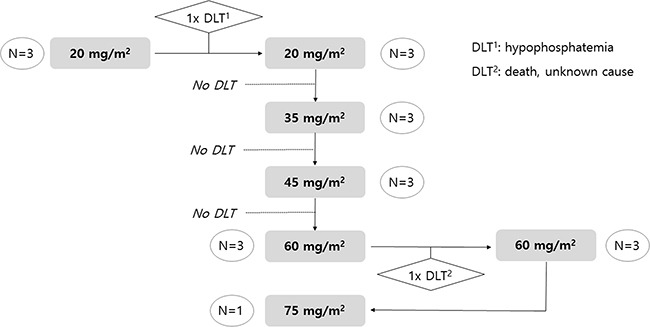
Dose scheme of PNP-DTX and result of enrollment

A total of 21 patients were screened, and 19 patients were treated from April 2010 to September 2011. Pharmacokinetic analysis, measurement of adverse events, and assessment of clinical outcome were evaluated in the treated 19 patients. The median age was 59 years (range, 42–65 years) and the number of woman was 8. The result of enrollment with dose-escalation scheme is depicted in Figure 4.

### The DLT and MTD

As planned, we first enrolled three patients into Group 1 (20 mg/m^2^), and there was a grade 3 hypophosphatemia case. Because there was no DLT in additionally enrolled three patients for the second cycle of group 1, dose escalation to group 2 was done. In Group 2 (35 mg/m^2^), there was no DLT. No DLT was observed in the three patients allocated to Group 3 (45 mg/m^2^) either. After moving to Group 4 (60 mg/m^2^), one of the three enrolled patients died with unknown cause, and another three patients were tested with the same dose level and successfully treated with 60 mg/m^2^ PNP-DTX. However, all six patients in Group 4 experienced grade 4 neutropenia, which was not a DLT because it was normalized within 7days. After stepping up to Group 5 (75 mg/m^2^), one case also suffered from grade 4 neutropenia without any experience of DLT. Enrollment was discontinued in the middle of Group 5 because we predicted inevitable severe neutropenia with Group 5 or greater, although it was not considered a DLT. Because the study was discontinued without predefined DLT in group 5 (75 mg/m^2^), the MTD could not be determined. However, 75 mg/m^2^ (group 5) was tentatively decided as the MTD of administered PNP-DTX.

### Adverse events and safety of PNP-DTX

Adverse events were observed in all of the 19 study patients at a total number of 230. Some of the adverse events were thought to be related to the test drug, PNP-DTX. The most prominent adverse events in each group were myalgia, neutropenia, dyspnea, fatigue, decreased appetite, and increased levels of aspartate or ALT. Most of the common drug-related adverse events included myalgia, neutropenia, fatigue, or decreased appetite. Among these adverse events, grade 4 neutropenia was the most common toxicity and developed in 10 (53%) patients. Grade 1 myalgia in eight (41%) patients and grade 1 alopecia in eight (41%) patients followed. In each dose group, the grade 3 or higher adverse events were as follows: grade 3 hypophosphatemia in one patient (17%) in Group 1 (20 mg/m^2^); grade 3 neutropenia in three (100%) patients and grade 3 anemia, asthenia, and headache in each patient in Group 2 (35 mg/ m^2^); grade 4 neutropenia in all three (100%) patients and grade 3 cataracts in one (33%) patient in Group 3 (45 mg/ m^2^); grade 4 neutropenia in all six (100%) patients and grade 3 syncope in one (17%) patient in Group 4 (60 mg/m^2^); and grade 5 of unknown origin in one (17%) patient; and grade 4 neutropenia in one (100%) patient in Group 5 (75 mg/m^2^). During the clinical trial, two patients died, from an unknown cause and pneumonia, respectively.

The ECOG performance scale did not change during the designated clinical trial in most patients except for one patient in Group 2 who showed an ECOG 2 after follow-up. An unexpected adverse physical examination; namely, changes in the nails, was observed in one 42-year-old female patient in Group 4. Changes in body weight or BSA were low in all of the study patients, and there was no change in drug administration dose due to significant BSA changes. The vital signs of the patients did not significantly change during or after the clinical trial in any groups.

### Pharmacokinetics

**Figure 5:**
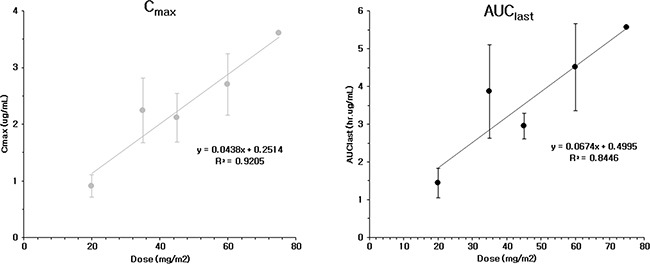
C_max_ and AUC_last_ of PNP-DTX from phase I clinical study

A total of 285 plasma samples was donated to enable pharmacokinetic analysis. The DTX, M2 and M4 metabolite level were measured. There was a greater than 2-fold increase in the C_max_ and AUC_last_ by escalating the drug dose from Group 1 (20 mg/m^2^) to Group 2 (35 mg/m^2^), but a more gradual increase was observed after a dose of 35 mg/m^2^. (Figure 5) The CL and V_d_ values did not differ between each dose group, but there was a minimal value of 35 mg/m^2^ in each group. The pharmacokinetic parameters of each dose group are listed in Table 1. Renal clearance was not measured because its value was expected to be that of the parent compound, DTX. The C_max_ of PNP-DTX (60 mg/m^2^) was comparable to that of DTX (75 mg/m^2^), and the AUC of PNP-DTX (45 mg/m^2^) was also comparable to that of DTX (75 or 80 mg/m^2^). The terminal t_1/2_ was longer for PNP-DTX (15.42–20.81 h) than for DTX (7.4–13.69 h).

**Table 1:** Pharmacokinetic (PK) parameters of docetaxel (DTX) from plasma data on day 1 of PNP-DTX injection

PK parameter		Dose of PNP-DTX (mg/m^2^/d)					
		Dose	20	35	45	60	75
		Group	1	2	3	4	5
	No. of enrolled patients		6	3	3	6	1
C_max_ (mg/L)		Mean	0.91	2.24	2.11	2.70	3.60
		SD*	0.20	0.57	0.43	0.54	
AUC_last_ (hr·μg/L)		Mean	1.44	3.87	2.95	4.51	5.57
		SD*	0.39	1.24	0.34	1.15	
AUC_inf_ (hr·μg/L)		Mean	1.59	4.09	3.14	4.82	5.77
		SD*	0.51	1.24	0.33	1.21	
V_d_ (L)		Mean	727.56	391.08	667.12	688.00	547.66
		SD*	449.84	257.18	156.86	438.23	
CL (L/hr)		Mean	23.31	14.56	23.71	22.32	24.62
		SD*	4.71	5.60	3.94	5.55	
t_1/2β_ (hr)		Mean	20.81	17.70	19.39	20.43	15.42
		SD*	9.64	6.51	1.68	7.47	

* SD: Standard deviation

### Clinical Outcomes

Eighteen of the study patients were assessed for a response, because a patient died before the second treatment cycle. Two patients showed PR (12%). Eight (44%) patients were assessed as having SD and the other 8 (44%) patients showed progressive disease (PD). Patients showing PR were allocated to Group 4 (60 mg/m^2^) in which the objective response rate was 40% (2/5 patients). For the maximal response, PR was achieved in 4 (23%) of 18 patients. Six patients (33%) showed SD and 8 patients (44%) showed PD. (Table 2)

**Table 2:** Tumor response to PNP-DTX in each patient

Group (Dose)	No	Primary Ds.	Response after 2^nd^ cycle	Maximal Response
1 (20mg/m^2^)	11	Colon	PD	PD
	12	Colon	PD	PD
	13	Rectum	SD	SD
	14	Colon	SD	SD
	15	Colon	SD	SD
	16	Colon	PD	PD
2 (35mg/m^2^)	21	Colon	PD	PD
	22	Cervix	PD	PD
	23	Colon	PD	PD
3 (45mg/m^2^)	31	Breast	SD	PR
	32	Bladder	SD	SD
	33	Colon	PD	PD
4 (60mg/m^2^)	41	Breast	SD	SD
	42	Adrenal	PD	PD
	43	NSCLC	N/A*	N/A*
	44	Breast	PR	PR
	45	Bladder	PR	PR
	46	Kidney	SD	SD
5 (75mg/m^2^)	51	Pancreas	SD	PR

* Not-available because patient died by unknown cause after 1^st^ cycle of PNP-DTX PR: Partial remission, SD: Stable disease, PD: Progression of disease

## DISCUSSION

Peripheral neurotoxicity and hypersensitivity are well-known non-hematological toxicities of DTX. Hypersensitivity is induced by surfactant, polysorbate 80, to contain anhydrous DTX [7]. Corticosteroids are routinely pre-treated to decrease the incidence of hypersensitivity, and to minimize peripheral neurotoxicity by DTX, but these toxicities are still barriers to high doses or the prolonged use of DTX.

Investigators expected that the selective higher concentration of DTX to only tumor cells might increase the efficacy and decrease the toxicities at the same dose [5, 7–10]. PNP is a challenging drug delivery system to improve the clinical pharmacodynamics of chemotherapeutic agents by enhancing drug permeability to tumor [5]. We developed a PNP-bound DTX formulation and already reported that PNP-DTX was more cytotoxic than DTX in A549 lung cancer cell line and that the pharmacologic activity of DTX was preserved in PNP-DTX [6]. Nab-paclitaxel, under the trade name Abraxane (Celgene, NJ), is the most famous and successful nanoparticle (NP) drug containing taxane. Albumin is bonded to paclitaxel as a delivery vehicle to overcome the poor water solubility of paclitaxel [11]. Abraxane is approved for use in the treatment of breast, lung, and pancreatic cancers. Albumin or protein is currently the most powerful way to formulate a taxane-binding NP delivery system, but the use of albumin as a base material sometimes make it hypersensitive by itself. Our material, PNP-DTX, causes no hypersensitivity. A few other NP drug launched recently; BIND's targeted ACCURINS and CriPec-Docetaxel (brief information from http://cristaltherapeutics.com/programs/cripec-docetaxel/) from Cristal Therapeutics [12]. The basic concept that they try to make more effective delivery of payloaded DTX to targeted solid tumor is the same with Abraxane or PNP-DTX. Our drug might have simpler drug structure than ACCURINS, and could have the equivalent efficacy to tumor with CriPec-DTX in lower concentration. However, due to the limited source of data about two drugs mentioned above, intensive comparison should be in the future.

From the data of preclinical studies, PNP-DTX was more cytotoxic than free DTX especially in orthotopic mouse model, even if its potency was not so higher than that of DTX. According to the knowledge about the EPR effect, the effect of NP drug might be higher in subcutaneous model than in orthotopic model [13]. However, experimental results from our preclinical studies were not accord with theoretical expectation. We repeated the same experiments in subcutaneous and orthotopic mouse models under the same condition to confirm the initial results, and preclinical results were not changed. We don't clearly understand that there is another acting pathway of PNP-DTX in addition to the EPR effect. However, we could confirm that PNP-DTX continued to preserve its cytotoxic effect of preloaded DTX and have a little more efficacy than free DTX.

From the data of phase I clinical study, the maximal response rate (CR or PR) in all of the evaluable patients was 22% (4/18 patients). Most responses were observed at 45 or 60 mg/m^2^, which is lower than conventional dose of DTX, 75 mg/m^2^ or higher. The MTD was tentatively determined to be 75 mg/m^2^ and the pharmacokinetics of PNP-DTX were stable at different plasma concentrations. The AUC of 45 mg/m^2^ PNP-DTX has been considered comparable to the 75 or 80 mg/m^2^ for DTX, and C_max_ was comparable to 60 mg/m^2^ of DTX. There was a case of grade 5 toxicity, but the cause of death was unknown. Relation to test drug might be possible but not directed. The most common toxicity was grade 3–4 neutropenia, but it was recovered within 7 days and not counted as DLT as defined. Nail disorder is common toxicity of DTX, but only a patient developed it in this study [14]. Two patients died during trial. A patient died immediately after the 1^st^ cycle of PNP-DTX and considered relation with treatment. The other patient died from pneumonia, but it was not considered drug-related because there was no neutropenia at that time. In summary, PNP-DTX had the same pharmacokinetic profile in lower dose than that of free DTX. The same efficacy at decreased dose of payloaded DTX might logically contribute to lowering the rate of neurotoxicity or hypersensitivity, but we could not yet confirm because of short follow-up and limited number of patients. We are preparing the study design of phase II clinical trial for advanced pancreatic cancer and/or other type of cancer to prove this promising result.

In conclusion, the PNP-DTX had more potent cytotoxic effect than DTX, especially in preclinical orthotopic mouse model. The MTD of PNP-DTX could not be confirmed, but 75 mg/m^2^ was tentatively determined. The PNP-DTX of 45 mg/m^2^ had the same pharmacokinetic profile with that of 75 mg/m^2^ DTX. Objective response rate over PR was 22% and most common toxicity was grade 4 neutropenia, which is not a DLT. Further step clinical study would be designed.

## MATERIALS AND METHODS

### Preparation of PNP-DTX

We prepared PNP by using polylactic acid monovalent salt (PLA-COONa) and mPEG-PLA as a copolymer [15]. Ethanol-solubilized DTX were mixed with a polymeric matrix of mPEG-PLA and PLA-COONa in ethanol. Calcium chloride was added to fabricate PNP-DTX and the next step was a filtering process followed by lyophilization. Transmission electron microscopy (TEM) and dynamic light scattering were used to characterize the shape, size and size distribution of PNP-DTX.

### Preclinical study in a subcutaneous and orthotopic mouse model

The human primary pancreatic adenocarcinoma cell line, BXPC3, was prepared for the preclinical *in vivo* study. We performed all of the experiments within the guidelines of the protocol reviewed by Institutional Animal Care and Use Committee of Asan Institute for Life Science.

For the subcutaneous model, BXPC3 cells (1×10^6^ cells) were subcutaneously implanted into the right hind legs of BALB/c nude mice. The mice with tumors measuring 100 mm^3^ were randomized to control, DTX (10 mg/kg), and PNP-DTX (10 mg/kg) groups. There were five mice in each group. We administered each drug twice on days 0 and 7. Each length of X, Y, Z was measured using caliper and tumor volume was calculated.

For the orthotopic model, BALB/c nude mice were also used. Luciferase-transfected BXPC3 cells (1×10^6^ cells) were surgically implanted into the pancreas of the mice and an *in vivo* imaging system was used. To compare the efficacy of PNP-DTX with those of the standard drug, we randomly divided surgically implanted mice into five groups: control, DTX (10 mg/kg), gemcitabine (50 mg/kg), PNP-DTX (10 mg/kg), and PNP-DTX plus gemcitabine. Four mice were examined in each group. Each drug was injected intravenously once on day 0. Tumor visualization was accomplished by performing luminescence imaging on day 10, 16 and 24. Mice were intraperitoneally injected with D-luciferin (0.3 mg/head; no. P/N 122796; PerkinElmer Inc.), after 10 minutes placed in IVIS Spectrum (PerkinElmer Inc.) for acquirement of whole body luminescence imaging. The region of the interested (ROI) was measured with the radiance (photons/s/cm2/sr) using analysis program, Living Image 4.4 (Caliper Life Sciences, PerkinElmer Inc.). The tubulin polymerization assay was done to visualize the acting mechanism of PNP-DTX. Tumor tissues isolated from mice were fixed in 4% formaldehyde overnight. Paraffin-embedded tumor tissue was sectioned in 3 μm, mounted on saline-coated slides, deparaffinized, and rehydrated in a graded alcohol series. The tissue was blocked with 5% bovine serum albumin (BSA) in PBST (0.01% Triton X-100 in PBS) and incubated with anti-alpha-Tubulin (1:200; no. 2125; Cell Signaling Technology, Inc.) diluted in PBST overnight at 4°C. On the following day, the tissue was washed in PBST and incubated with Alexa Fluro^®^ 488 donkey anti-rabbit (1:500; Jackson ImmunoResearch Laboratories, Inc.) for 2 hours at room temperature, and counterstained with Vectashield mounting media containing DAPI (Vector Laboratories).

### Patient eligibility for phase I clinical study

This was a single-center, prospective, open-label phase I clinical trial that allowed us to determine the MTD, and evaluate the safety and pharmacokinetics of PNP-DTX in advanced solid tumors. It was fully approved by the Institutional Review Board of Asan Medical Center (Seoul, Korea), and written informed consent was obtained. All stages of the clinical trial were performed in accordance with clinical practice guidelines. Inclusion criteria were an age above 18 years; prior informed consent; advanced stage solid tumors that were pathologically confirmed and validated by Response Evaluation Criteria in Solid Tumors (RECIST) criteria or an imaging modality; advanced disease that was resistant to any known chemotherapeutic agent; treatments such as chemotherapy, immunotherapy, or radiotherapy apart from initiation of the drug under investigation; Eastern Cooperative Oncology Group (ECOG) performance of 0 to 1; expected survival of over 3 months; and appropriate hematological, renal, and liver function on screening including hemoglobin (Hb) ≥10 g/dl, absolute neutrophil count (ANC) ≥ 1.5×10^9^/L, platelet count ≥ 100×10^9^/L, total serum levels of bilirubin ≤ 1.5 mg/dL, aspartate aminotransferase (AST) and alanine aminotransferase (ALT) ≤ 2.5 x upper normal limit (UNL), alkaline phosphatase (ALP) ≤ 2.5 x UNL, and creatinine ≤ 1.5 x UNL.

Exclusion criteria encompassed a surgical history within 2 weeks of screening; history of metastasis to the central nervous system; sensory or motor neuropathy above a National Cancer Institute-Common Terminology Criteria for Adverse Events (NCI-CTCAE) grade 2; history of resistance to DTX treatment; hypersensitivity to the drug under investigation or diluting agent; history of severe heart problems within 6 months; uncontrolled active infectious disease; history of participating in other clinical trial within 4 weeks of screening; pregnancy or breast-feeding; no consent to participate in the clinical trial; and use of contraception from screening to 3 weeks after the clinical trial.

### Drug Administration for phase I clinical study

The primary purpose was to determine the MTD of PNP-DTX. Secondary purposes were to evaluate the DLT, safety, its pharmacokinetics, and objective response rate. All of the patients went through a designated screening laboratory test after informed consent was given. Volunteers fit for clinical study were educated to visit the clinic every 3 weeks after drug injection for evaluation.

The cycle of drug administration and evaluation was 3 weeks, and PNP-DTX was injected intravenously for 1 hour on day 1 in each cycle. Minimum 2 cycles of administration were necessary for response. The patients visited the outpatient clinic every 3 weeks, and the follow-up evaluation was 3 weeks after PNP-DTX which stopped. Patients having a complete response (CR), partial response (PR), or stable disease (SD) received a continued prescription after the clinical trial free of charge, if it was deemed by the clinician to be beneficial.

The body surface area (BSA) was calculated using Mosteller formula I and recalculated before each cycle administration by BSA (m^2^)=([height (cm) x weight (kg)]/3600)^1/2^ [16].

### Dose group, DLT and MTD

We came up with the dose scheme with 20 mg/m^2^ as starting dose. Planned dose groups were 35 mg/m^2^, 45 mg/m^2^, 60 mg/m^2^, 75 mg/m^2^, and 90 mg/m^2^. Allocated number of patient in each group was 3 at first. Dose could be escalated without any DLT in each group. If more than 2 patients experienced any DLT, the study should be stopped. Whereas, if there was DLT in only a patient, three more patients should be added to current dose group. If more than 2 of totally 6 patients experienced DLT, the study also should be stopped and the MTD would be determined as a step lower dose group.

The DLT was defined as follows; grade 3 or higher hypersensitivity to drug despite any premedication before administration, any grade 3 or higher non-hematological toxicity except nausea and vomiting, or hematological toxicities like neutropenia <0.5×10^9^/L over 7 days, febrile neutropenia <1.0×10^9^/L with 38.5°C or higher body temperature, infectious neutropenia <1.0×10^9^/L, platelet <25.0×10^9^/L, or platelet 50.0×10^9^/L accompanying bleeding or requiring platelet-transfusion.

### Pharmacokinetic evaluation

The area under the curve in a plot of the drug concentration in the blood plasma against time (AUC_last_, AUC_inf_) of DTX was calculated. The maximal concentration (C_max_), time to reach C_max_(T_max_), elimination half-life (t_1/2_), clearance (CL), and volume of distribution (V_dss_) were measured or calculated descriptively as the average and standard deviation.

### Efficacy and safety

The longest diameter of measurable tumor in the axial plane on CT was measured and recorded at baseline and response evaluation. Tumor response was determined by RECIST criteria ver. 1.1 and any patients showing CR, PR at the maximal response were considered the responding group [17]. The ECOG performance status (ECOG-PS) [18], physical examination, vital signs, laboratory data, and toxicity were recorded to evaluate the safety of PNP-DTX administration. CTCAE version 4.0 was adopted to categorize the toxicities.

### Statistical considerations

Pharmacokinetic parameters were calculated by WinNonlin Professional (version 6.1, Pharsight Co.) and descriptive data (mean, standard deviation and variation coefficient) of each patient were obtained. Non-compartmental analysis was done using linear up and log-down methods. The objective response was mainly calculated in the intention-to-treat group and additionally per protocol population. The confidence interval was 95% in all patient groups and dose groups.
